# Treatment of the Fluoroquinolone-Associated Disability: The Pathobiochemical Implications

**DOI:** 10.1155/2017/8023935

**Published:** 2017-09-25

**Authors:** Krzysztof Michalak, Aleksandra Sobolewska-Włodarczyk, Marcin Włodarczyk, Justyna Sobolewska, Piotr Woźniak, Bogusław Sobolewski

**Affiliations:** ^1^Physics Faculty, Laboratory of Vision Science and Optometry, Adam Mickiewicz University in Poznań, Umultowska Street 85, 61-614 Poznań, Poland; ^2^Nanobiomedical Center of Poznań, Umultowska Street 85, 61-614 Poznań, Poland; ^3^Department of Biochemistry, Medical University of Lodz, Mazowiecka Street 6/8, 92-215 Łódź, Poland; ^4^Outpatient Clinic, Polish Mother's Memorial Hospital-Research Institute, Rzgowska Street 281/289, Łódź, Poland

## Abstract

Long-term fluoroquinolone-associated disability (FQAD) after fluoroquinolone (FQ) antibiotic therapy appears in recent years as a significant medical and social problem, because patients suffer for many years after prescribed antimicrobial FQ treatment from tiredness, concentration problems, neuropathies, tendinopathies, and other symptoms. The knowledge about the molecular activity of FQs in the cells remains unclear in many details. The effective treatment of this chronic state remains difficult and not effective. The current paper reviews the pathobiochemical properties of FQs, hints the directions for further research, and reviews the research concerning the proposed treatment of patients. Based on the analysis of literature, the main directions of possible effective treatment of FQAD are proposed: (a) reduction of the oxidative stress, (b) restoring reduced mitochondrion potential Δ*Ψ*_m_, (c) supplementation of uni- and bivalent cations that are chelated by FQs and probably ineffectively transported to the cell (caution must be paid to Fe and Cu because they may generate Fenton reaction), (d) stimulating the mitochondrial proliferation, (e) removing FQs permanently accumulated in the cells (if this phenomenon takes place), and (f) regulating the disturbed gene expression and enzyme activity.

## 1. Long-Term Adverse Reactions Caused by Fluoroquinolones

Fluoroquinolones (FQ) belong to the group of broad-spectrum antibiotics, effective for both gram-negative and gram-positive bacteria. The most frequently prescribed drugs are ciprofloxacin (CIP), norfloxacin (NOR), and levofloxacin (LEV). FQs employ their antibacterial effect by preventing bacterial DNA from unwinding and duplicating which takes place by inhibition of bacterial topoisomerase and gyrase. For the last three decades, FQs played an important role in treatment of serious bacterial infections, especially hospital-acquired infections. However, due to the possibility of serious side effects, these drugs are not currently first-line medicines and their use becomes more restrictive and limited. FQs should be reserved for those who do not have alternative treatment options.

In 2016, the US Food and Drug Administration (FDA) updated warnings, using next “black box” for oral and injectable FQs. The authors showed that FQs, when used systemically, are associated with disabling and potentially permanent serious side effects. These side effects can involve the disruption of tendons, joins, muscles, nerves, nervous system disturbances, and even induction of type 2 diabetes. Due to the increasing number of reports about FQ toxicity and long-term complications, FDA has introduced significant restrictions on their use in recent years, particularly in children and in people aged 65 years.

### 1.1. Tendon Rupture

FQs are associated with a significant risk of tendonitis and tendon rupture. Stephenson et al. [1] showed in their review the incidence of tendon injury among those taking FQs to be between 0.08 and 0.2%. In 2014, Lewis and Cook [2] proved that FQ-related tendinopathy is a complication of treatment with this family of antibiotics and it is usually linked with 1 or more synergistic factors: male sex, age, renal disease, rheumatic disease, coprescription of corticosteroid, and physical activity. For this reason, some sport medicine specialists have advised avoidance of FQs for athletes. Some authors, for example, [3, 4], proved that chronic renal disease, concomitant use of corticosteroids, and age > 60 years are known risk factors for FQ-induced tendinopathies. Concluding, FQs are associated with an increased risk of tendinitis and tendon rupture. This risk is further increased in those over age 60, in kidney, heart, and lung transplant recipients, and with use of concomitant steroid therapy.

### 1.2. Nervous System Disturbances

Taking FQs is associated with their neurotoxicity as well [5–8]. The main symptoms being correlated to FQ treatment include insomnia, restlessness, and, rarely, seizure, convulsions, and psychosis [9–11]. Many reports point to chronic persistent peripheral neuropathy to be generated by FQs [12–18]. Cohen [19] showed that a possible association between FQ and severe, long-term adverse effects involving the peripheral nervous system as well as other organ systems is observed.

### 1.3. Cardiotoxicity

Stahlmann and Riecke [20] showed that FQs prolong the heart's QT interval by blocking voltage-gated potassium channels. In some cases, this can be a life-threatening condition because prolongation of the QT interval can lead to torsades de pointes, a life-threatening arrhythmia. Statistically, significant risk factors for clinically significant changes in QTs were hypokalemia and a left ventricular ejection fraction < 55%.

### 1.4. Hepatotoxicity and Nephrotoxicity

The other adverse reactions generated by FQs include hepatotoxicity [21] and nephrotoxicity [22]. Golomb et al. [23] reported a case-series study and showed the potential occurrence of serious, persistent, and delayed multisymptom serious side effects apparently triggered by FQ use causing severe functional compromise and disability in previously vigorous, healthy individuals. In this study, Golomb et al. described patients who developed new-onset symptoms during and following FQ use. Domains of serious and persistent sequels included the better-recognized tendon and muscle issues but extended to the well-reported but still often unappreciated potential for cognitive, psychiatric, peripheral nervous, and gastrointestinal issues as well as endocrine issues.

### 1.5. Diabetes Mellitus

Telfer [24] conducted interesting study about FQ intake and development of type 2 diabetes mellitus (T2DM). They hypothesized that FQs induce an intracellular Mg^2+^ deficit that can lead to insulin resistance. Their data suggests that FQ exposure predisposes an individual to develop diabetes. He also showed a strong correlation between the increase in FQ application in the US in years 1990–2012 and the increase in T2DM incidences in subsequent years which suggests a large part of T2DM to be maybe generated by FQ exposure.

### 1.6. FQ-Associated Disability

In 2016, Kaur et al. [8] conducted basic science and clinical investigations of a newly identified adverse drug reaction, termed FQ-associated disability (FQAD). They proved that severe toxicities that develop when cancer patients receive supportive care drugs such as FQs are important, yet difficult to understand, detect, and to communicate to clinicians. Their findings supported recommendations of the FDA's advisory committee. Revision of FQ-product labels should be considered to include prominent descriptions of a newly identified FQ-associated long-term toxicity.

Concluding, patients with impairments of the CNS (e.g., epilepsy or arteriosclerosis), prolongation of the QT, elderly persons, and individuals with concurrent use of glucocorticoids or chronic renal diseases should not be treated with FQs. FQs are contraindicated in children because they cause destruction of the immature joint cartilage in animals. The use in pediatrics is restricted to life-threatening infections.

## 2. Oxidative Stress

One of the main effects generated by FQs in cells is connected with the oxidative stress (OS). Thus, a brief review about OS is presented below.

The main aspect of OS involves the overdosed leakage of electrons from the electron transport chain (ETC). In the normal, healthy state, the Krebs cycle (KC) supplies hydrogen from glyco- and lipolysis in the form of NADH_2_ and FADH_2_ from KC to ETC. ETC separates hydrogen into protons and electrons. Protons are transported into mitochondrial intermembrane space (IMS) generating proton gradient, and mitochondrial membrane potential Δ*Ψ*_m_ across the inner membrane and electrons is transported into oxygen. This complex process is a masterwork of the evolution because 4 electrons must enter the oxygen simultaneously in order to produce 2 water molecules. In the case of the block in the electron transport and the oxygen not to be fully reduced in one step, the reactive oxygen species (ROS) are created, which might be able to generate OS.

The total physiological electron leakage earlier estimated to be about 1% of electrons supplied to ETC [25, 26] currently has been estimated in favourable conditions to be 0.1–0.5% in rest [27–29] and 0.01–0.03% in the exercise [30, 31]. Assuming the oxygen consumption by the human body in the rest to be about 500 g (which corresponds the basal metabolic rate of 1920 kcal/24 h), the total electron current in all human mitochondria can be easily estimated to be about 70 Amperes. The 0.1% leakage denotes the leakage electron current (LEC) to be about 0.07 Amperes. In the case of physical or mental exercise, both the metabolic rate and LEC increase several times; however, recent studies show that LEC does not depend so strictly on the metabolic rate [30]. Disturbing the precise mechanism of electron flow through ETC causes nearly always the increased leakage which can increase LEC even up to 10% (~7 A in the rest). Majority of toxins joining ETC may do this leak. Because the increased OS is the side effect of the exercise state and OS is one of the limits of the exercise, the increase in LEC during the rest may reduce the reserve for the OS increase during the exercise. The patient may feel tired even during small physical or mental exercise.

O_2_^−^ is the first oxygen radical created by LEC. Joining the second electron causes the generation of H_2_O_2_, and joining the third electron to H_2_O_2_ creates a very dangerous hydroxyl radical OH^∗^. H_2_O_2_ is relatively stable and works as a cellular sensor of the OS state and is involved in the cellular metabolism regulation [30, 32]. Brand [30] has recently detected 11 sites in ETC where electrons leak from ETC generating OS. Brand and coworkers propose new postulate that the rate of electron leak does not depend on the electron flow rate but on the redox state (electron pressure) of the given site of leakage. Thus, ETC blockers increase the leak in sites before the block and decrease the leak behind the block. Majority of the sites generate the leak to the mitochondrial matrix and only two sites to both sides of inner mitochondrial membrane: the sites III_Qo_ connected with complex III and G_Q_ connected with glycerol 3-phosphate dehydrogenase [30]. Brand et al. suggest also that the increase in O_2_^−^ production from complex III that signals hypoxia to the HIF-1*α* signaling system in cells is probably caused by indirectly changed metabolite concentrations that deliver more electrons to the ETC and leads to higher electron leak to oxygen. This is of high importance because as will be discussed later in this paper, HIF-1*α* signaling is blocked in FQ patients disturbing strongly very important energy production regulatory pathway.

The last radical being created during the OS is OH^∗^. It is very dangerous because its lifetime is very short (10^−9^ s) and the cell does not possess any enzymes removing it. The overdosed production of OH^∗^ is generated especially by Fenton reaction or in the ischemia state in which, because of lack of the oxygen, all the electrons create ROS. This massive ROS production induces the death of the ischemic cell.

The relatively high physiological LEC forced the evolution to create the mechanisms carrying against free radicals. The first and, thus, most important barrier is the enzyme SOD2 (MnSOD, mitochondrial superoxide dismutase) which annihilates O_2_^−^—the first molecule of the O_2_-radical chain (2O_2_^−^ + 2H^+^ → O_2_ + H_2_O_2_). H_2_O_2_ is next removed by catalase or glutathione peroxidase. Meanwhile, it comes out of mitochondria and regulates, for example, redox-dependent Kv1.5 channels in the cell membrane [32]. This mitochondrion-ROS-Kv channel axis is now recognized as basis of an important O_2_-sensing mechanism in many tissues [33].

**Figure 1:**
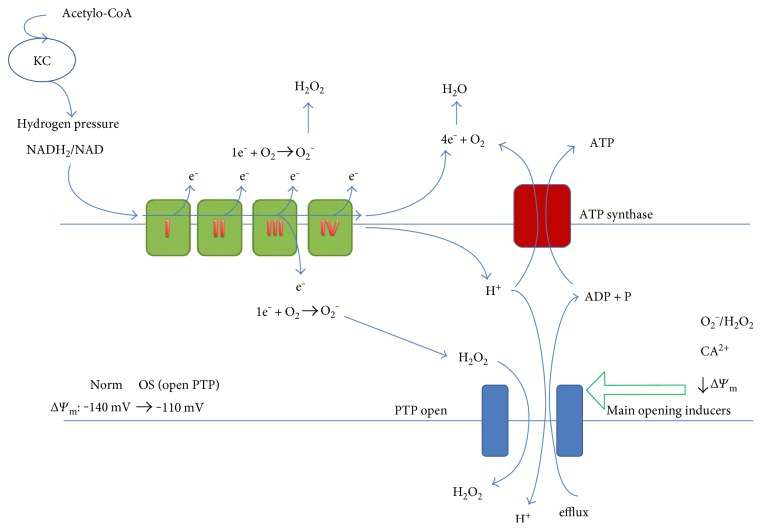
The schematic presentation of the ATP production system. The acetyl-CoA supports Krebs cycle to produce NADH_2_. Hydrogen from NADH_2_ (and FADH_2_) enters the cytochrome chain. Some electrons leak before they reach oxygen generating O_2_^−^ and next H_2_O_2_. H_2_O_2_ comes away from mitochondria and works as a redox signaling molecule. The amount of leaking electrons depends mainly on NADH_2_/NAD ratio (hydrogen pressure). The degree of PTP opening must be precisely regulated and depends on many factors, for example, O_2_^−^/H_2_O_2_, Ca^2+^, and Δ*Ψ*_m_.

The simplified scheme of the electron leakage from ETC is presented in Figure 1.

### 2.1. The Role of Mitochondrial Permeability Transition Pores (PTP) in the Regulation of Energy Production

The mitochondrial permeability transition pore (PTP) is a large protein complex placed in the outer mitochondrial membrane being precisely regulated by many factors [34, 35]. It consists mainly of voltage-dependent anion channel (VDAC), adenine nucleotide translocase (ANT), and cyclophilin D (CypD). In order to make the cell function properly, the degree of the opening state of this complex must be precisely fitted to the actual physiological state of the cell. If the complex is open, the nonselective traffic between the IMS and cytosol of small-charged particles, water, and substances up to 1.5 kDa takes place. ADP can enter mitochondria to produce ATP but the protons leak from IMS to cytoplasm reducing the mitochondrial potential Δ*Ψ*_m_ from −140 mV to about −110 mV and contributing to apoptosis. If the complex is closed, ADP cannot enter mitochondria to produce ATP and Δ*Ψ*_m_ growths from −140 to −160 mV. The decrease in Δ*Ψ*_m_ is a characteristic for OS state, and the increase in Δ*Ψ*_m_ is a characteristic for some types of cancers [32]. This observation explains the ability of FQs to treat cancers [36–41].

The main factors that regulate PTP state [34] are as follows:

- Opening PTP
  - [Ca^2+^]_mit_: concentration of mitochondrial Ca^2+^
  - Reduced Δ*Ψ*_m_ (positive loop)
  - Free radicals (oxidative stress)
  - Inorganic phosphate (only with Ca^2+^)
  - Some apoptotic factors
- Closing PTP
  - Acidic pH (a part of cancer state is lactate accumulation in the cell)
  - ATP, ADP and NADH
  - Mg^2+^

Hexokinase II (HKII), mitochondrial creatine kinase (CK), benzodiazepine receptor (PBR), and Bcl-2-family members (Bcl-2, Bcl-xL, and Bax) are putative regulatory components.

**Figure 2:**
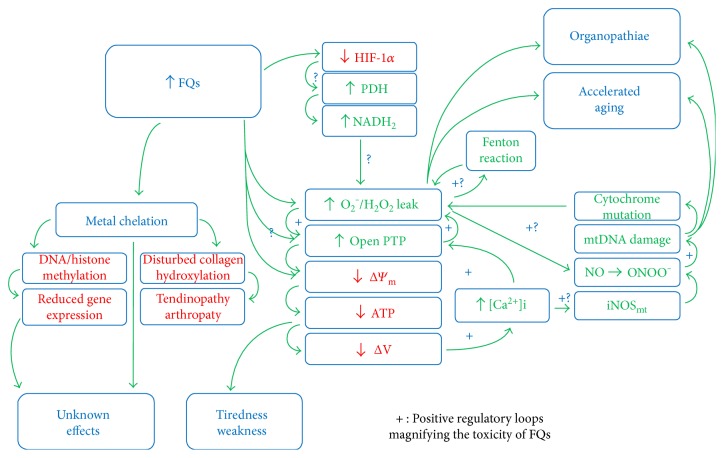
The main ways of FQ toxicity. The positive regulatory loops magnifying the toxicity of FQs are marked with “+.” The “?” signs denote the possible but not confirmed effects of FQ toxicity.

It can be observed that OS contributes to opening PTP and reducing Δ*Ψ*_m_. Reduced Δ*Ψ*_m_ causes further opening of PTP and the decrease in energy production. The final step and physiological sense of this positive loop are the induction of the apoptosis. However, if the apoptosis induction is not reached, the new balance between factors being in the common regulatory loop finds a new equilibrium point which can be far away from that optimal one. The “positive loop” regulation of PTP points to its high sensitivity to different factors controlling it and suggests the regulation of PTP to be one of the most important points of energy production control. Some positive regulatory loops are presented in Figure 2. According to the control theory, such positive loops denote that the reaction to small stimulus may be strongly magnified. The influence of FQs on the detailed regulation of PTP is the urgent topic for further research.

### 2.2. How the Cell Adapts to OS State

Increased OS state is characterized first by increased H_2_O_2_ level in the cell. In the physiological state, H_2_O_2_ level informs the cell and nucleus about the mitochondrial energy production state because, in the physiological conditions, it is connected with the metabolic rate of the cell. In the case of disturbed metabolic regulation (e.g., mitochondrial toxins), increased OS and open PTP, ATP production is reduced. The main process that consumes ATP in the cell is the Na/K pump that removes Na^+^ from the cell, pumps K^+^ to the cell, and generates the negative membrane potential of the cell. The work of Na/K pump is the most fundamental process of life because it consumes 20% to 50% of ATP and generates strongly negative charge inside the cell. Many other transports that take place across the cell membrane work as Na^+^ cotransport, for example, amino acids, phosphates, Ca^2+^, and boric acid. One molecule of Na^+^ cotransport contributes to about 30% of ATP/ADP Gibbs free energy; thus, it can be treated as “small energy bricks” when compared to ATP as a large one. The lack of ATP reduces the work of Na/K pump reducing negative membrane potential ΔV and K^+^ concentration in the cell. However, H_2_O_2_ generated during OS activates the opening of redox-dependent Kv1.5 channels in the cell membrane [32] contributing to the further efflux of K^+^ from the cell. The escaping K^+^ increases temporarily the negative potential inside the cell. It can be concluded that restoring the negative cell potential is more important for the cell than keeping high K^+^ concentration in the cell.

On the other hand, in the case of reduced ATP production, the lowered ΔV opens voltage-gated Ca^2+^ channels which causes Ca^2+^ influx into the cell [32]. Increased [Ca^2+^]_i_ activates calcineurin. Activated calcineurin shifts NFAT (nuclear factor of activated T-cells) to the nucleus where it inhibits Kv1.5 potassium channel production. Lowered amount of Kv1.5 channels reduces K^+^ efflux. Concluding, in the OS state, Kv1.5 channels are more open; however, their amount is reduced. Two opposite mechanisms regulating the membrane potential and K^+^ efflux find some new equilibrium. This mechanism seems to be the natural adaptation process to the increased metabolic rate of the cell generating physiological OS. In the state of increased metabolic rate, the concentration of K^+^ (and most probably Mg^2+^) is reduced, the concentration of Ca^2+^ and H_2_O_2_ (and most probably Na^+^) is higher, and the membrane potential is also reduced. If the healthy cell returns from the increased metabolic rate to the resting state, all these parameters return to their optimal values. This return depends on the ability of the mitochondria to produce ATP which drives Na/K pump.

In the case of the permanent OS or disturbed state of PTP being inadequately open, this return cannot take place because the ATP production is lowered. The cell being in the resting state comes to the state which can be called “permanent stress adaptation.” In the case of the necessity to increase the metabolic rate, further increase in metabolic rate is difficult because of the lack of physiological adaptation reserve. The final effect for the patient is the feeling of “the lack of energy.” Many other regulative processes take place, of course, as OS adaptation. However, the above-described changes belong in authors' opinion, to the primary regulatory axes.

## 3. Molecular Mechanisms of FQ Toxicity

Good understanding of the OS state is very important for understanding the consequences of OS generation by FQs. From the therapeutic point of view, the important question concerns the molecular mechanisms leading FQs to generate OS, because they determine the possible effective treatment of this state. Many people are waiting for the understanding of the FQ toxicity mechanisms and treatment methods. Beside OS, epigenetic effects of FQs are of high importance as well. The epigenetic effects may depend on the methylation of DNA and/or histones; however, ROS contribute also to epigenetic changes [42]. Some authors point also to the similarity of bacterial and mitochondrial DNA, both existing in circular super-twisted helices and gyrase-like enzymes being postulated to be responsible for the organization of mitochondrial DNA, suggesting the possible direct effect of FQs to mitochondrial DNA leading to the disturbed mitochondrion regeneration and division [43, 44]. The changes in the cytoskeleton were observed also after FQ treatment [45], and cytoskeleton has been demonstrated to be strictly connected with energy dissipation and organization in mitochondria [46–49]. The most important elements of FQ toxicity are presented in Figure 2. The positive regulatory loops magnifying the toxic effects are marked with “+.” Let us analyze the most important aspects of the molecular activity of FQs in the cell, being reported until now.

### 3.1. Chelating Bivalent Cations and Proteins by FQs

**Figure 3:**
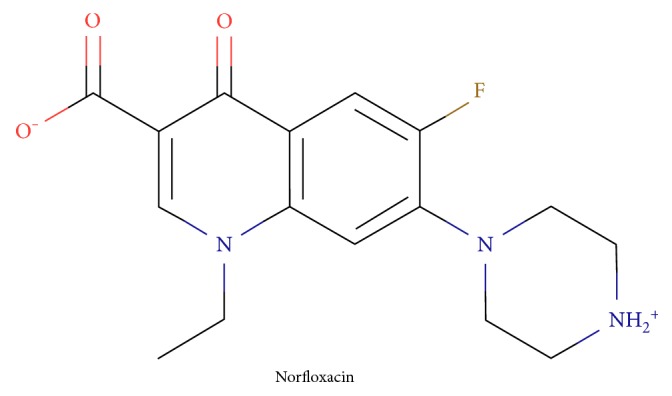
The exemplary FQ—norfloxacin and its zwitterion structure.

FQs are the group of chemical compounds called zwitterions. Zwitterion is a neutral molecule with both positive and negative electrical charges on its opposite sides. This feature makes them possible to create strong complexes with both protein anions and positively charged bivalent cations. The protonation constants for the acidic and base part of the FQ molecules were estimated as pK_1_ = 8.2–8.5 and pK_2_ = 5.6–6.2. These values denote that the dissociation coefficients are equal to about 90–95% in neutral intracellular conditions of pH = 7.0 for both the acidic and base side of the molecule [50]. The structure of the exemplary FQ is presented in Figure 3.

FQs possess two main sites for metal chelate formation. The first one, represented by the carbonyl and carboxyl groups in neighboring positions, is the most common coordination mode in the quinolone chelates [50]. Quinolones bind divalent cations as Mg^2+^, Ca^2+^, Cu^2+^, Zn^2+^, Fe^2+^, and Co^2+^, forming chelates with 1 : 1 or 1 : 2 metal : FQ stoichiometry or trivalent cations (e.g., A1^3+^ and Fe^3+^), forming chelates with 1 : 1, 1 : 2 or 1 : 3 metal : FQ stoichiometry. The constant values for CIP chelates have been measured to decrease in the following order: Al^3+^ > Fe^3+^ > Cu^2+^ > Zn^2+^ > Mn^2+^ > Mg^2+^ [51]. For NOR chelates, the relation is quite similar: Fe^3+^ > Al^3+^ > Cu^2+^ > Fe^2+^ > Zn^2+^ > Mg^2+^ > Ca^2+^. Let us observe that presented research [50, 51] did not analyze Se^2+^ ions which are also of high importance in the cell, because Se^2+^ is the cofactor of glutathione peroxidase removing H_2_O_2_. The examination of the ability of FQs to generate Se^2+^-FQ complexes is the important aim for further research.

Seedher and Agarwal [52] analyzed the ability of 5 cations: Fe^3+^, Al^3+^, Zn^2+^, Cu^2+^, and Mg^2+^ to create the complexes with four FQs and human serum proteins using fluorescence UV absorption spectroscopy. They measured the association constants to be of the order of 10^2^–10^4^ for the FQ-Me^n+^ interaction. The interaction was the highest for Al^3+^ and lowest for Mg^2+^. At a Me^n+^/drug ratio of 1 : 1, approximately 50%–73% of metal ion was bound per mole drug in most cases. Seedher's results indicate that chelate formation with bivalent metals can cause significant alteration in the human serum-FQ-binding affinity.

Koga [53] measured the FQ concentration ratio between intra- and extracellular fluid in human polymorphonuclear leukocytes using high-performance liquid chromatography. This c_i_/c_out_ ratio oscillated between 2.2 to 8.2. In another paper, Pascual et al. [54] presented c_i_/c_out_ gradient for trovafloxacin (TRV) to be about 9 for extracellular concentrations ranging from 0.5 to 25 ug/mL. Assuming net charge of the FQ molecules being close to 0, the expected ratio for the soluble fraction is expected to be close to 1 : 1. These results point to the ability of FQs to create complexes with different intracellular molecules which do not participate to the soluble fraction of FQs in the cell.

The other important feature of FQs has been presented by Andriole et al. [55]. Namely, they estimated the minimum solubility of FQs in neutral pH. They pointed that this class of molecules is characterized by very high melting point, generally > 200°C, which indicates that the crystal forms are very stable.

All these FQ features strongly support the thesis that FQs can survive in the cell for a long time contributing to chronic, long-term adverse reaction in patients treated with FQs. The question, to what extent this phenomenon takes place and if it contributes to chronic symptoms of FQAD, remains unclear.

Metal ion chelating seems to be the most fundamental feature of FQs which probably leads to all other observed toxic effects. The antibacterial effect is connected with chelating Mg^2+^ which disturbs the gyrase and topoisomerase interaction with DNA. However, the Mg^2+^ is described to create weaker chelates with FQs than other important ions like Fe^2/3+^, Cu^1/2+^, Zn^2+^, and Mn^2+^.

The well-described activity of FQs which concerns the Fe^3+^ chelating was examined by Badal et al. [56]. They examined that three FQs, NOR, CIP, and enrofloxacin (ENR), are the powerful Fe^2/3+^ chelators comparable with deferoxamine, a clinically useful Fe-chelating agent. He showed that Fe^2/3+^chelating by FQs leads to epigenetic effects through inhibition of *α*-dependent dioxygensases (DOXG) that require Fe as a cofactor. Three important DOXGs were analyzed: jumonji domain histone demethylase, TET DNA demethylase, and collagen prolyl 4-hydroxylase. The activity of all 3 enzymes was reduced by FQs in micromolar concentrations leading to accumulation of methylated histones and DNA and to inhibition of proline hydroxylation. The IC_50_ concentrations for Fe-chelation were equal to about 52 ± 20 uM for CIP, 44 ± 15 uM for NOR, 41 ± 20 uM for ENR, and 360 ± 25 uM for deferoxamine. These results point to high ability of FQs to absorb Fe^2/3+^ and reduce the activity of enzymes which use Fe^2/3+^ as a cofactor. The results suggest also the possibility that the tendon FQ toxicity is dependent on lack of collagen proline hydroxylation which changes significantly the mechanical properties of the collagen. Some other studies report, however, the association of the enhanced extracellular matrix metalloproteinases [51, 52], mainly collagenases expression [53], to be associated with FQ-induced tendinopathy.

It must be pointed that cytochromes are the other important proteins which use Fe^2/3+^ in hem groups as a cofactor. Thus, the question arises if the reduced free Fe^2/3+^ concentration in the cell contributes to ETC inhibition, electron leakage, and/or OS. The question arises, as well, which cellular effects are connected with Cu^2+^ and Zn^2+^ ions, because Cu^+^ is also an important cytochrome cofactor and Zn^2+^ is important due to high number of enzymes (about 300 total) being cofactored by this element and the total cytoplasm concentration of Zn^2+^ is similar and even higher than that of Fe^2/3+^. Valko et al. [57] present that redox-inert zinc (Zn^2+^) is the most abundant metal in the brain and an essential component of numerous proteins involved in biological defense mechanisms against oxidative stress. The depletion of zinc may enhance DNA damage by impairing DNA repair mechanisms.

Some effects of Mg^2+^ chelation are also well described, especially with respect to the cartilage damage. Mg^2+^ is an important intracellular ion being a cofactor of about 300 enzymes. Shakibaei et al. [58] showed that effects of individual dose of ofloxacin give identical effects in cartilage observed in electron microscopy as Mg^2+^-deficient diet suggesting that quinolone-induced arthropathy is probably caused by a reduction of functionally available Mg^2+^ in cartilage. Similar results were observed during cultivation of chondrocytes in Mg-free medium [45, 59–61]. The supplementation of Mg^2+^ accompanying the FQ treatment restored to some degree the cartilage lesions [60, 61], however, did not restore the reduced cell division [61]. This suggests that other mechanisms are involved in cell division reduction after FQs [44]. Mg^2+^ deficiency in immature dogs induced similar clinical symptoms as quinolone treatment: distinct alterations in chondrocytic fibronectin staining and their ultrastructure [62, 63]. The effect of Mg^2+^ supplementation had a two-way effect on FQ-treated cultured chondrocytes: the number of cells adhering to culture support was increased and cell morphology was comparable to that of control cells. This suggests that addition of Mg^2+^ restores extracellular Mg^2+^-dependent intercell interactions.

On the other hand, dietary Mg^2+^ is also presented to reduce intestinal FQ absorption [64–70] and it is also postulated to be in relation to diabetes type 2 and FQ treatment [24].

Summing up, the number of enzymes possessing reduced activity due to their ion-cofactor chelation is probably long and it is the important topic for further research. The separate problem consists the chronicity of ion chelation by FQs. The presented research does not describe the chronic state of FQAD but the phenomena taking place during FQ application. It must be analyzed as to which degree persistent ion chelation takes place at FQAD patients.

### 3.2. Oxidative Stress Generated by FQs

Many papers point to the feature of FQs to generate oxidative stress in the cells. The molecular mechanisms leading to OS state differ probably in details for different FQs depending on different abilities to chelate subsequent metal ions and on possible different abilities to change enzyme activity in the ion-independent manner.

An example of ion-independent FQ activity was presented by Qin and Liu [71]. They analyzed the influence of CIP and ENR on the erythrocytic catalase (CAT), a vital enzyme involved in OS reduction (CAT reduces H_2_O_2_ to O_2_ and H_2_O). The cellular tests firstly confirmed an enhanced oxidative stress in FQ-treated erythrocytes in the form of the GSH content depletion and decrease in CAT activity with CIP effect to be more harmful than ENR. Next, spectroscopic computations showed the FQ-binding places to CAT takes place mainly through electrostatic forces. Binding of two FQs not only caused the conformational and microenvironmental changes of CAT but also inhibited its molecular activity, which was consistent with the cellular activity measurements. On the other hand, the treatment with danofloxacin [72] increased antioxidant enzyme activities such as superoxide dismutase (SOD) and catalase (CAT), suggesting that the ability of subsequent FQs to change the activity of different antioxidative enzymes can vary significantly.

Many papers present the existence of OS induced by FQs, for example, Pouzaud et al. [73] measured the redox status change of immortalized rabbit tendon cells as a response to pefloxacin, ofloxacin, LEV, and CIP. All FQs showed moderate cytotoxicity on tendon cells after 24 h and more severe, significant toxicity after 72 h. The intracellular redox potential has been reduced slightly but significantly after 72 h even at concentrations 1 uM (~10 uM is the therapeutic one) and strongly reduced at concentrations 1 mM (100x higher than therapeutic ones). ROS production has increased slightly (~25%) but significantly at therapeutic conditions and strongly (~150%) at 100 uM (10x higher than therapeutic ones). The intracellular GSH concentration was reduced by 20–50% even at 0.01 uM concentrations (1000x smaller than therapeutic ones), and the collapse (decrease in 50–90%) in GSH was observed at 1 mM. The question arises if the GSH depletion is connected only with the increased H_2_O_2_ generation or also with a reduced activity of GSH-reductase which restores the reduced form of GSH (GSSG + NADPH_2_ → 2GSH + NADP).

It is important to observe that the rapid increase in the toxicity takes place after the given concentration of FQs is reached which is only slightly higher than the therapeutic one. The substantial increase in the ROS production which can lead to serious consequences begins at concentrations being approximately 10x higher than that of the therapeutic ones. This factor is, however, estimated with low precision because no intermediate concentrations were examined between 0.01, 0.1, 1, 10, 100, and 1000 uM. Assuming that some people are charged with reduced ROS annihilation ability (e.g., as presented in [21]), this toxicity limit may occur at lower, therapeutic concentrations. This experiment stays also in the agreement with clinical observations that the FQAD takes place especially in patients who were treated with higher FQ doses, by a longer period or with FQ series repeated a few times in a short period of time.

In the other paper, Pouzaud et al. [74] observed in vitro in rat tendons the increased ROS production and reduced GSH concentration. Similar results were also showed by Yu et al. [72].

Some papers point to detailed FQ effects on different enzymes. Hsiao et al. [21] found that TRV-induced OS on heterozygous SOD2 (+/−) deficiency mice was higher than on the normal mice. Hepatic protein carbonyls were increased by 2.5-fold, and hepatic mitochondrial aconitase activity was decreased by 20% in mutant, but not in wild-type mice. Because aconitase is a major target of peroxynitrite, they determined the extent of nitrotyrosine residues in hepatic mitochondrial proteins (peroxynitrite ONOO^−^ id formed by the combination of O_2_^−^ and NO). TRV significantly increased nitrotyrosine in SOD2 (+/−) mice only. TRV increased also the production of mitochondrial NO in immortalized human hepatocytes. Similarly, mitochondrial Ca^2+^ was increased by TRV, suggesting Ca^2+^-dependent activation of mitochondrial NOS activity. Furthermore, the transcript levels of the mtDNA-encoded gene Cox2/mtCo2 were decreased in SOD2 (+/−) mice only, while the expression of nDNA-encoded mitochondrial genes was not significantly altered in both genotypes, suggesting selective effects on mtDNA expression. These data indicate that TRV enhances hepatic mitochondrial peroxynitrite stress at increased basal O_2_^−^ levels, leading to the disruption of critical mitochondrial enzymes and gene regulation.

Another important information can also be found in [21]. OS generation by FQs may take place at lower FQ concentrations in some people being charged with lower ability to reduce OS. The reasons of reduced OS barrier may be different; however, the most important reasons seem to be the heterozygous mutations, trace element deficiencies, and charge of the cells with other toxins contributing to OS.

Kumbhar et al. [75] reported gatifloxacin (GAT) to produce retinal damage in rabbits and significant alteration in the antioxidant status indicated by the decreased activity of superoxide dismutase and decreased levels of blood glutathione with a concomitant increase in the activity of catalase, glutathione peroxidase, and glutathione S-transferase enzymes. The levels of malondialdehyde were also elevated. The effects were dose-dependent. Talla and Veerareddy [76] examined the OS parameters in the blood after CIP, LEV, and GAT therapy on SOD3 (extracellular), glutathione, plasma antioxidant status, and lipid peroxides evaluated at 53 patients on different dosage regiments up to 5 days. The significant elevation of lipid peroxide was observed in patients treated with CIP and LEV. The substantial depletion in SOD3 and glutathione was observed especially in CIP patients. All three FQs reduced the plasma antioxidant status, but especially CIP and LEV.

Liu et al. [77] determined the effect of ENR on the release of lactate dehydrogenase (LDH), reactive oxygen species (ROS), superoxide dismutase (SOD), total antioxidant capacity (T-AOC), malondialdehyde (MDA), mitochondrion membrane potential (Δ*Ψ*_m_), and apoptosis in the hepatic cell line of grass carp. The doses of 50, 100, and 200 ug/mL increased the LDH release and MDA concentration, induced cell apoptosis, and reduced the Δ*Ψ*_m_ compared to the control. The highest dose of 200 ug/mL also significantly reduced T-AOC.

All the above-presented experiments show the increased OS state after FQ treatment. The changes in enzyme activity vary between experiments, tissues, and kinds of FQs suggesting possible variability of the common relations. What seems to be important is the reduction in SOD activity which is the first-line barrier against O_2_^−^. New experiments must estimate these changes in details; however, more attention must be paid to mitochondrial MnSOD which cares against mtDNA damage by O_2_^−^ generated by leaking electrons from ETC to mitochondrial matrix. The increased activities of some anti-OS enzymes seem to be the OS adaptation processes.

### 3.3. Reduction of Mitochondrial Δ*Ψ*_m_ Potential by FQs

One of the cellular symptoms present in the FQ-charged cells is the reduction of the mitochondrial potential Δ*Ψ*_m_ [37, 38, 77–79]; however, the detailed mechanism of this phenomenon remains unknown. Since the main mitochondrion uncoupling factor is the PTP, the reasons of the reduced Δ*Ψ*_m_ should be searched between factors that regulate PTP opening. The first possibility is the OS by itself being generated by FQs. ROS can induce VDAC oligomerization (the main part of PTP) to yield a megachannel creation which are postulated to create large holes being able to release cytochrome c to cytoplasm. O_2_^−^-induced apoptosis has been found to be inhibited by DIDS or anti-VDAC antibodies (VDAC blockers), which suggests that O_2_^−^ increases VDAC-dependent permeabilization of the outer mitochondrial membrane [80, 81].

One of the proteins which can support PTP opening is translator protein (TSPO), called also peripheral-type benzodiazepine receptor or isoquinoline-binding protein. TSPO is predominantly located on the surface of the mitochondria where it is postulated to physically associate with VDAC-ANT. It has been suggested that TSPO may activate PTP opening, causing Δ*Ψ*_m_ reduction and leading to apoptosis [80, 81].

Some authors suggest that epileptogenic activity of FQs possibly relates to GABA-like structure of some FQs which may allow them to act as GABA antagonists [82, 83]. Since TSPO is also a benzodiazepine receptor, similar interaction may maybe also take place between FQs and TSPO leading to opening PTP.

The problem of the PTP opening is probably, however, more complicated and it is in relation with many other factors involved in energy production and apoptosis induction in the cell. The broad reviews about regulation of PTP and VDAC (the main PTP protein) are presented in [34, 35].

The other way to reduce Δ*Ψ*_m_ is the opening of UCP channels which generate the proton leak through the inner mitochondrial membrane. UCP regulation is a separate problem and possible way for treating FQAD patients. The review of this problem is published by Divakaruni et al. [84].

### 3.4. Does Fenton Reaction Take Place in FQAD Patients?

The next problem that is connected especially with Fe^2+^ and Cu^+^ ions is the possibility of the Fenton reaction (FR) to be generated on Fe^2/3+^ and Cu^1/2+^ ions, but maybe on the FQ-Fe^2+^ complexes as well. Fenton reaction consists in the conversion of O_2_^−^ and H_2_O_2_ into strongly dangerous OH^∗^ radical; it takes place on Fe^2+/3+^ and Cu^1+/2+^ ions and consists of 2 steps:

1. Fe^2+^ + H_2_O_2_ → Fe^3+^ + OH^∗^ + OH^−^
2. a. Fe^3+^ + O_2_^−^ → Fe^2+^ + O_2_ or (2) b. Fe^3+^ + H_2_O_2_ → Fe^2+^ + O_2_^−^ + 2 H^+^

This reaction increases strongly the effects of OS in the cell and if it is too intense, it may contribute to cell death. The question arises as to what degree does the Fenton reaction magnify initial OS effects.

There is no evidence that would prove such reaction to take place in FQAD patients; however, four theses could be postulated for further research: (a) The reaction is increased just due to the increased substrate concentration (O_2_^−^ and H_2_O_2_) for FR. (b) The reaction intensity is reduced with respect to that expected due to the reduced Fe^2+^ level in the cell. This can take place due to reduced membrane potential and reduced ability Fe^2+^ to be pulled into the cell. (c) The reaction is magnified due to the increased ability of FQ-Fe^2+^ complexes to generate OH^∗^ in FR reaction by itself. (d) The reaction is magnified due to upregulation of Fe-transporting protein which increases Fe^2+^ concentration in the cell. Similar upregulation has been detected in *Streptococcus pneumoniae* by Ferrandiz and de la Campa [85, 86]. They observed the upregulation of the genes of the fat DCEB operon involved in iron (Fe^2+^ and Fe^3+^) uptake. In accordance, they observed an attenuation of LEV lethality in iron-deficient media. However, the bacterial gene regulation cannot be directly compared to that mammalian one. On the other hand, electro-Fenton reaction is described to perform degradation of LEV in experimental conditions [87, 88]; however, it seems to be of low probability for such reaction to take place at in vivo conditions.

### 3.5. Changes in Gene Expression and Enzyme Activities after FQ Treatment

Besides OS aspects connected with FQAD, some papers point to other effects of FQ toxicity. Fox et al. [89] measured reverse-transcriptase quantitative polymerase chain reaction analyses on total RNA isolated from supraspinatus tendon of rats. They showed the significant upregulation of IL-1b mRNA, tumor necrosis factor (TNF), matrix metalloproteinases MMP-3 (30x increase), MMP-13 (7x), and the tissue inhibitor of metalloproteinases- (TIMP-) 1 (4x) in the FQ-treated rats. FQ-treated groups showed significantly less fibrocartilage and poorly organized collagen at the healing enthesis compared with control animals.

Aranha et al. [41] measured the gene expression effects of CIP on prostate carcinoma and healthy control cells. Treatment of prostate cancer cells with CIP resulted in a dose- and time-dependent inhibition of cell growth (70–100% with 50–400 ug/mL). Cells were arrested at the S and G2/M phases, and apoptosis was induced. The cyclin-dependent kinase (CDK) inhibitor p21/WAF1 was downregulated 12 h following CIP in treatment which can lead to rapid CDK2 activation and caspase-induced apoptosis. There was also observed significant increase in the Bax/Bcl-2 ratio with translocation of proapoptotic Bax to mitochondria and activation of caspase-3. Let us recall that Bax, Bcl-2, and capsace-3 are involved in PTP opening state.

Badal et al. [56] showed that NOR, CIP, and ENR as iron chelators lead to epigenetic effects through inhibition of alpha-ketoglutarate-dependent dioxygenases that require iron as a cofactor. Three dioxygenases were examined in HEK293 cells treated with FQ. At micromolar concentrations, these antibiotics inhibited jumonji domain histone demethylases, TET DNA demethylases, and collagen prolyl 4-hydroxylases, leading to the accumulation of methylated histones and DNA and inhibition of proline hydroxylation in collagen. These effects may explain FQ-induced nephrotoxicity and tendinopathy. Let us observe that the changes in DNA and histone methylation state cause strong and broad epigenetic effects being difficult to predict.

Liang et al. [90] measured the subchronic toxic effects of NOR on a swordtail fish by measuring mRNA expression of cytochrome P450 1A (CYP1A), cytochrome P-450 3A (CYP3A), glutathione S-transferase (GST), P-glycoprotein (P-gp), and their corresponding enzyme activities. Results showed that NOR significantly affected the expression of CYP1A, CYP3A, GST, and P-gp genes in swordtails. The gene expressions were, however, more responsive to NOR exposure than their corresponding enzyme activities. The analyzed enzymes are very important because they express the ability to catalyze detoxification of xenobiotic substrates, including FQs. The possible reduction of its activity in humans may be of high importance, because FQs undergo biotransformation in the liver from approximately 50 percent for pefloxacin to about 6 percent for ofloxacin [91]. Although glucuronide conjugates have been identified as minor metabolites for some agents, most metabolic reactions involving quinolones occur through microsomal oxidative mechanisms at the cytochrome P-450 site. These metabolic alterations involve the piperazinyl moiety and usually result in compounds with significantly less microbiologic activity than the parent drugs. However, the conclusions from fish cannot be directly transferred to humans and the results suggest the possibility of the delayed toxicity to be connected with reduced detoxification induced by itself.

Similar results of P-450 inhibition by FQs were found in chicken by Shlosberg [92] and Granfors et al. [93]. Regmi et al. pointed to the inhibiting effect of FQs in dogs on P-450 1A but not on P-450 3A [94, 95].

### 3.6. FQs and HIF-1*α*

Dioxygenase inhibition by FQs was predicted by Badal et al. [56] to stabilize transcription factor HIF-1*α* by inhibition of the oxygen-dependent hypoxia-inducible transcription factor prolyl hydroxylation. In dramatic contrast to this prediction, HIF-1*α* protein was eliminated by FQ treatment. Badal and coworkers explored this effect to be caused by inhibition of HIF-1*α* mRNA translation.

The natural function of HIF-1*α* system is to change the metabolism of the cell into the anaerobic pathway in order to protect the cell against OS. The conversion of pyruvate to lactate is enhanced by upregulation of the lactate dehydrogenase-A (LDH-A). On the other hand, pyruvate dehydrogenase complex is inhibited by HIF-1*α* by upregulating pyruvate dehydrogenase kinase (PDK) which inhibits PDH-A. Thus, HIF-1*α* inhibits pyruvate to enter KC and to produce NADH_2_. As presented by Brand et al. [96], NADH_2_/NAD ratio (which can be called “hydrogen pressure”) is the main factor determining the escape of electrons from the ETC chain, making this process to high degree independent of the total ETC electron flow. (The term “hydrogen pressure” reflects the natural similarity of ETC to the water flowing through the pipe with small holes. The water escaping through the holes depends on the pressure but not on the water velocity in the pipe.) Thus, in case of the lack of the oxygen in the tissue which is the final recipient of the electrons from NADH_2_, HIF-1*α* turns on the side way for hydrogen, not to put it into ETC and produce OS but to produce lactate. The lack of this safety valve (HIF-1*α*) in FQ patients may cause the shift of the overdosed amounts of hydrogen into ETC causing overdosed electron leak. Assuming this phenomenon to be important in FQ-treated patients, the glyco- and lipolysis inhibitors could be considered as OS reductors. The natural glycolysis inhibitor in the diet is citric acid which inhibits phosphofructokinase (the main regulatory point of glycolysis) and activates fructose 1.6 bisphosphatase which catalyzes the opposite reaction promoting gluconeogenesis and pentose cycle pathway. On the opposite, the vinegar (acetic acid) could be contradicted by FQAD patients because it promotes glyco- and lipolysis both contributing to possible overdosed “hydrogen pressure” and OS.

## 4. Therapeutic Conclusions

The treatment of the FQAD, especially that lasting for many years, is a very difficult therapeutic problem. The effectiveness of different therapies carried out on patients is rather low. A large number of patients suffers from chronic tiredness, tendinopathies, neuropathies, and lack of sleep, even more than 12 h/24 h. Understanding all the molecular mechanisms of the FQ activity in the cell is the urgent aim for the current science to find methods helping these people.

The main question that arises here concerns the reasons of chronicity of FQAD symptoms which last for many years, sometimes, even after a standard 5-day FQ treatment. 3 reasons can be taken into consideration:

- Long-lasting OS destroys the mitochondrial DNA and the newly synthesized proteins creating cytochrome complexes are disturbed in their structure leading to permanent electron leakage and OS.
- The complexes of FQ with proteins and cations are so stabile that they exist in the cells by many years disturbing energy production and epigenetics.
- Epigenetic changes in gene regulation become persistent many years of FQ application even in the case of lack of FQ in the cell.

The answer, which of these three possible reasons contribute to the chronicity of FQAD symptoms, is of high importance with respect to the problem of effective treatment of this state. The research answering these questions must be performed as fast as possible.

In the case of mtDNA destroying, the treatment is difficult and it must focus on the stimulation of mitochondrial replication. The more destroyed mitochondria must be removed and the less destroyed must replicate in order to substitute for the removed ones and to reduce the total LEC. After many replications, the most healthy mitochondria would dominate the cell. The final effect would depend on the state of the most healthy mitochondrium in the cell. The second possibility is to increase the ratio of cell exchange in the given tissue. The cells with more destroyed mitochondria must be shifted to apoptosis while more healthy cells must substitute them. This process, however, cannot take place in the central nervous system and muscles because the cell exchange is close to zero in these tissues. Also, the collagen exchange is very low causing the tendon regeneration to be a difficult and long-lasting problem.

If new research would confirm the existence of FQs in the cells and mitochondria in the amounts making possible their permanent interactions with proteins and cations even after many years of FQ application, the research must focus on methods on how to remove FQs from strong protein and cation complexes. The simplest way seems to be the application of increased doses of metal cations Fe^2+^, Cu^+^, Mn^2+^, Zn^2+^, and Mg^2+^ which are natural FQ-competitors for protein-binding sites. It should be pointed that bivalent metal ions enter the cell to some degree due to the negative membrane potential of the cell and, next, enter the mitochondria due to the negative value of mitochondrial potential Δ*Ψ*_m_. The Nernst equation ΔV = RT/zF ln(*c*_in_/*c*_out_) defines the equilibrium between the ion concentration gradient and voltage across the membrane. If the membrane voltage ΔV/Δ*Ψ*_m_ decreases, the concentration equilibrium gradient *c*_in_*/c*_out_ of bivalent ions decreases significantly as well, because the lowered potential may not be able to pull bivalent cations into the cell/mitochondrium up to the required concentration.  Table 1 shows the exemplary calculations.

Table 1 shows that the decrease in membrane voltage reduces strongly the ability of the cell to pull X^2+^ ions into the cell/mitochondrium. The question arises as to what extent the transport of X^2+^ ions into the cell is disturbed in FQAD patients. And next, if the possible lower X^2+^ concentration depends only on reduced ΔV/Δ*Ψ*_m_ or maybe also on the disturbed membrane transport being blocked by FQs joining to metal-binding sites of transport proteins? Every analyzed bivalent cation requires a separate analysis.

The third possible reason of the permanent FQAD state is the permanent disorder in gene expression caused by some positive loop regulations. For example, reduced Fe^2+^ level disturbs oxoglutarate-dependent dioxygenases and increases methylation of DNA and histones which leads to reduced Fe^2+^ absorption to the cell. Many other loops are possible which could cause the chronic state of the patient despite the lack of FQs in the cell. They have to be recognized in order to find the methods restoring the normal regulatory state. This case is the most hopeful for patients, because, for example, the mtDNA damage is rather difficult to be effectively treated and gene expression regulation is difficult, however, possible.

Until detailed knowledge concerning FQ toxicity would be recognized, the following directions in supporting FQAD patients are proposed according to the known and probable mechanisms of FQ toxicity:

- Reduction of the oxidative stress: assuming that H_2_O_2_ is not effectively removed from the cell after FQ treatment, subsequent consequences may occur such as opening Kv1.5 channels, Fenton reaction, peroxynitrite radical creation, opening PTP channels, decoupling mitochondrial potential, and, finally, breaking down OS barrier. The detailed comparison of different FQs with respect to OS is urgent to determine which FQs are safer in use and which ones are more dangerous. Reduction of OS is a very broad area. There are thousands of natural substances which possess the antioxidant capacity and which are able to reduce free radicals leaked from ETC. One should, however, remember that they work as one to one. It means that, as a rule, they are not reduced in the cell after free radical annihilation in order to work in cycle. They only reduce the size of free radical damage.

Among the antioxidants which enter easily, the mitochondria are the most interesting ones. Lowes et al. [79] show that the mitochondrion-targeted antioxidant MitoQ protects against fluoroquinolone-induced oxidative stress and mitochondrial membrane damage in human Achilles tendon cells. In cells treated with MitoQ, the oxidative stress was lower and mitochondrial membrane potential was maintained.

Simonin et al. [97] report oxidative damage of collagen I to be prevented by coadministration of N-acetylcysteine (150 mg/kg) to the mice. Tsai et al. report similar anticytotoxic effect of resveratrol [98]. Vitamins C and E belong also to this group; however, after they annihilate some radicals, they can be reduced using NADPH_2_. Some papers point to the ability of vitamin E to reduce the consequences of FQ-induced damage [99]. Vitamin C is presented to possess the ability to protect against lethal gamma photon irradiation in mice—a strong source of OS [100]. Trace elements Zn^2+^, Cu^1/2+^, Se^2+^, Fe^2/3+^, and Mn^2+^ are cofactors of important antioxidative enzymes. Selenium supplementation is reported [101] to partially restore oxidative stress and sperm damage in FQ-treated cells.

Mn^2+^ seems to be very important, because it is a cofactor of mitochondrial SOD2 being the first barrier against O_2_^−^ and carrying mtDNA against free radical damage. Thus, the amount of trace elements in the cell must be satisfactory. Detailed research is required for Fe^2/3+^ and Cu^1/2+^ in order to find if their supplementation does not increase Fenton reaction to occur. Citrate and other glycolysis inhibitors may reduce the “hydrogen pressure” on ETC reducing to some degree LEC and OS.

(b) Restoring reduced mitochondrial potential Δ*Ψ*_m_: restoring the reduced mitochondrial potential may be one of the important steps in restoring the proper regulatory balance in FQ-patients; however, it is not a trivial task. On the one hand, reducing OS may contribute to restoring Δ*Ψ*_m_; on the other hand, the reasons of PTP opening seem to be more composed and require advanced research. The points of interest may be reactivating HIF-1*α* system, reducing intracellular and intramitochondrial Ca^2+^ concentrations, restoring membrane potential ΔV, and restoring intracellular Mg^2+^, all contributing to PTP closing. Cyclosporine A and metformin are postulated to be able to close PTPs [102, 103] and protect against OS [102, 104–106]; thus, it is an interesting substance for possible FQAD treatment.
(c) Supplementation of uni- and bivalent cations that are chelated by FQs: the role of uni- and bivalent cations was partially discussed in point A. Additionally, the role of Mg^2+^ and K^+^ must be presented. Both ions possess high intracellular concentrations (Mg^2+^: 20x and K^+^ 40x higher than extracellular). K^+^ is probably removed from the cell in the OS state by opening redox-sensitive Kv1.5 channels. Mg^2+^ is strongly chelated by FQs, but, probably, it also escapes from the cells due to some unrecognized mechanisms. The supplementation must take into consideration the regulatory effect of kidney, which removes overdosed amounts of both cations to the urine. Thus, small but often doses are rather recommended in order to keep a bit higher concentrations of both ions in the blood plasma, which makes it possible to reduce the concentration gradients across the cell membranes and facilitate entering both to the cell.
(d) Supporting the mitochondrial replication in the cell—pulling more damage to apoptosis and proliferation of the more healthy ones: supporting the mitochondrial exchange (removing that destroyed ones and replication of that more healthy ones) is the necessary way in the case of irreversible mtDNA damage. The substance that is postulated to possess the ability to promote the mitochondrial biogenesis is pyrroloquinoline quinone (PQQ) [107, 108]. This substance is also postulated to be OS protective [109].
(e) Removing FQs permanently accumulated in the cells (if this phenomenon takes place): the problem of FQ accumulation is purely present in the available research. Thus, first, it is the urgent topic for establishing if this phenomenon really, and to what extent, takes place. Removing accumulated FQs may undergo in two ways: by cytochromes P-450 in the microsomes and by different processes which can remove the molecule outside the cell. Activating the reduced cytochrome detoxification may be an important point in FQAD patients. On the other hand, ozonation has been described to be an effective method for removing the first generation FQ—flumequine from the liquid water [110]. Thus, ozone therapy can be examined to be a method of FQ degradation in the body.
(f) Regulating the disturbed epigenetics and enzyme activities: every factor presented above contributes to the disturbed gene expression which can contribute to vicious circle-like regulations causing the new regulatory balance lying far away from that optimal one. If it is the main reason of the chronic state of FQAD patients, then there is a big chance to find methods for quick and effective treatment of this state. However, the problem is of high complexity.

## Figures and Tables

**Table 1:** The conversion of Nernst equation *c*_in_/*c*_out_ = exp(ΔV zF/RT) for bivalent ions as Mg^2+^, Mn^2+^, Fe^2+^, and Zn^2+^ shows strong relation between the actual membrane potential and ability of the cell/mitochondrium to attract and absorb the ions into the cell/mitochondrium. Reduced membrane potential ΔV is a strong factor which hinders entering bivalent cations to the cell. However, the detailed mechanisms of individual ion transport must be analyzed.

ΔV/Δ*Ψ*_m_	Equilibrium *c*_in_/*c*_out_ gradient (*z* = 2 and *T* = 310 K)
−160 mV	160.000x
−140 mV	36.000x
−110 mV	3.800x
−90 mV	850x
−70 mV	190x
−50 mV	42x
−30 mV	9x
